# Evaluation of cholera surveillance systems in Africa: a systematic review

**DOI:** 10.3389/fepid.2024.1353826

**Published:** 2024-06-12

**Authors:** Kyeng Mercy, Ganesh Pokhariyal, Noah Takah Fongwen, Lucy Kivuti-Bitok

**Affiliations:** ^1^ Department of Medical Microbiology and Immunology, University of Nairobi, Nairobi, Kenya; ^2^ Division of Surveillance and Disease Intelligence, Africa Centres for Disease Control and Prevention, Addis Ababa, Ethiopia

**Keywords:** cholera, surveillance, evaluation, Africa, framework

## Abstract

**Introduction:**

Despite several interventions on the control of cholera, it still remains a significant public health problem in Africa. According to the World Health Organization, 251,549 cases and 4,180 deaths (CFR: 2.9%) were reported from 19 African countries in 2023. Tools exist to enhance the surveillance of cholera but there is limited evidence on their deployment and application. There is limited evidence on the harmonization of the deployment of tools for the evaluation of cholera surveillance. We systematically reviewed available literature on the deployment of these tools in the evaluation of surveillance systems in Africa.

**Method:**

Three electronic databases (PubMed, Medline and Embase) were used to search articles published in English between January 2012 to May 2023. Grey literature was also searched using Google and Google Scholar. Only articles that addressed a framework used in cholera surveillance in Africa were included. The quality of articles was assessed using the appropriate tools. Data on the use of surveillance tools and frameworks were extracted from articles for a coherent synthesis on their deployment.

**Result:**

A total of 13 records (5 frameworks and 8 studies) were fit for use for this study. As per the time of the study, there were no surveillance frameworks specific for the evaluation of surveillance systems of cholera in Africa, however, five frameworks for communicable diseases and public health events could be adapted for cholera surveillance evaluation. None (0%) of the studies evaluated capacities on cross border surveillance, multisectoral one health approach and linkage of laboratory networks to surveillance systems. All (100%) studies assessed surveillance attributes even though there was no synergy in the attributes considered even among studies with similar objectives. There is therefore the need for stakeholders to harmoniously identify a spectrum of critical parameters and attributes to guide the assessment of cholera surveillance system performance.

## Introduction

Cholera is a bacterial infection of humans caused by *Vibro. cholerae*, a gram-negative bacillus (1). The last five years, the world has seen a global rise in the number of cholera cases reported. In 2017 alone, over 1.2 million cases and 5,000 deaths were reported globally with Asia accounting for 84% of all cases reported (2). This is a more than six-fold increase compared to the number of cases reported in the year 2015. In 2022, over 80 000 cases and 1,863 deaths were reported from 15 countries on the continent and some of these occurred in the context of tropical storms and flooding. In the year 2023, a total of 251,549 cases and 4,180 deaths (CFR: 2.9%) were reported from 19 African countries in 2023. This represents a two-fold increase in the number of cases and deaths reported on the continent compared to 2022. All cases were reported from the Western, Central and Eastern and Southern Africa regions (3).

Apart from fatalities, cholera outbreaks have had far-reaching socioeconomic disruptions in the lives of populations. It was estimated in 2015 that, cholera cost households worldwide more than $20 million in out-of-pocket expenses, $8.5 million in public sector expenditures, and $12.1 million in loss of productivity due to morbidity while the loss of productivity due to untimely deaths were estimated at $985.5 (4, 5).

There have been several commitments globally and regionally aimed at flattening the cholera curve. Through these commitments countries have made significant efforts in strengthening their surveillance and response systems to detect and respond timely to cholera cases. In Africa, at least 47 countries have adapted the integrated disease surveillance and response strategy (IDSR) within which cholera is listed as one of the priority diseases for immediate reporting. This strategy has improved surveillance and response processes in these countries. In 2018, the WHO regional office for Africa developed a multisector monitoring framework aimed at achieving the milestones and objectives for cholera control set by the Global Task Force on Cholera Control [GTFCC (6)]. This framework was focused on the realization of the reduction of 90% of cholera deaths globally by 2030 as set by the GTFCC. Within this document, regular joint multisector monitoring, and evaluation of the status of the framework implementation was a critical recommendation to serve as a mechanism to identify best practices as well as gaps for the improvement of cholera prevention, surveillance, and response. It remains unclear if evaluations conducted in countries to assess the efficiency and effectiveness of surveillance and response to cholera are comprehensive enough to measure critical parameters and targets of a robust surveillance system.

The aim of this review was to assess existing surveillance tools and frameworks used in the evaluation of cholera surveillance systems and make recommendations for an enhanced surveillance evaluation framework. The specific objectives include identifying published frameworks for assessing cholera surveillance in sub-Saharan Africa and critically analyzing the gaps on the application of these frameworks in the assessment of cholera surveillance systems. Finally, following the gaps identified, we made recommendations on the improvement of cholera surveillance evaluation framework.

**Research questions:**

1. What are the frameworks and tools for assessing cholera surveillance system in sub-Saharan Africa?
2. What are the gaps in the application of these frameworks in the evaluation of cholera surveillance systems?
3. How can the gaps in surveillance be mitigated to improve the robustness of the evaluation of cholera surveillance systems?

## Methodology

We conducted a systematic review which followed the Preferred Reporting Items for Systematic Reviews and Meta-analyses reporting checklist [PRISMA (7)]. The protocol was developed and shared with three continental stakeholders for review and input. Our study focused on the evaluation of cholera surveillance systems in all 55 African Union Member States. We reviewed available surveillance evaluation frameworks and studies that applied the frameworks. We assessed studies that evaluated cholera surveillance systems using vertical and integrated approach. However, studies that focused on evaluating the integrated disease surveillance systems in countries where cholera outbreaks were not reported, were excluded. We assessed each study against a set of defined variables consolidated from the available existing tools used (Table 1). Expected outcomes of interest in this study was to understand the spectrum of variation of the different surveillance tools used in assessing cholera surveillance since 2012. We also explored how these tools complimented each other and proposed a set of recommendations for a more coordinated and comprehensive approach in cholera surveillance system assessments.

**Table 1:** List of reviewed studies characterized by parameters deployed in assessment.

Key Assessment constituents	Governance, legislation and policies				Core functions					Supportive function							Quality and timeliness
Name of article	Framework used	Legislation and policies	Cross-border collaboration	Information flow	Case/signal definitions (case detection)	Reporting tools (Case reporting)	Feedback mechanism	Verification and assessment	Lab capacity/confirmation	Data management analysis, interpretation & use	Guidelines and SOPs	Multi sectoral OH coordination	Resources	Workforce (Supervision and training)	Digitalization	Linkage of laboratory systems	System Attributes
Cholera public health surveillance in the Republic of Cameroon-opportunities and Challenges (2016) Ngwa et al. (8)	IDSR & WHO M & E framework	√	**X**	√	√	√	√	√	√	√	√	**X**	√	√	√	X	√
Evaluation of cholera surveillance system in Osu Klottey District, Accra, Ghana (2011–2013) Adjei et al. (9)	US CDC	X	X	√	√	√	√	√	√	√	X	X	√	√	X	X	√
A rapid assessment of the implementation of integrated disease surveillance and response system in Northeast Nigeria, [Luka et al. (10)]	IDSR	√	**X**	√	√	√	√	√	√	√	√	**X**	√	√	√	X	X
Evaluation of Cholera and Other Diarrheal Disease Surveillance System, Niger State, Nigeria-2012 [Bashorun et al. (11)]	US CDC	X	X	√	√	√	√	√	√	√	√	X	√	√	X	X	√
Evaluation of the integrated disease surveillance and response system for infectious diseases control in northern Ghana [Nyaaba et al. (12)]	IDSR	X	X	√	√	√	√	√	√	√	X	X	√	√	√	X	X
Evaluation and use of surveillance system data toward the identification of high-risk areas for potential cholera vaccination: a case study from Niger [Guerra et al. (13)]	US CDC	X	X	X	√	X	X	X	X	√	X	X	X	X	X	X	√
Assessment of the cholera surveillance system in the Ledzokuku-Krowor municipality in the greater Accra region of Ghana [Achempem, (14)]	US CDC	X	X	X	√	√	X	√	X	√	X	X	√	√	X	X	√
Evaluation of integrated disease surveillance and response (IDSR) core and support functions after the revitalisation of IDSR in Uganda from 2012 to 2016 [Masiira et al. (15)]	IDSR	√	X	√	√	√	√	√	√	√	√	X	√	√	√	X	√

X, absent; √, present.

### Definition of keywords

**Surveillance:** In the literature, “surveillance” is “the ongoing, systematic collection, analysis, interpretation, and dissemination of data regarding cholera for use in developing preventive actions to reduce morbidity, mortality, and to improve health” (16).

**Performance assessment:** a systematic process that seeks to monitor, evaluate, and communicate the extent to which various aspects of a system meet its key objectives”.

### Eligibility criteria

Articles in any format including editorials, reviews, and original research and abstracts of relevant articles addressing cholera surveillance evaluation in Africa from 2012 to 2023 and published frameworks and tools for communicable disease surveillance systems in Africa were included in the study. On the other hand, articles whose objectives did not include the evaluation of cholera surveillance system in Africa as well as non-English articles, (unless an English abstract was available) and articles that were not accessible or did not include enough information for extraction of the study variables were excluded from the study.

### Search strategy (data sources and literature search)

We searched four electronic databases, including PubMed, Medline, Google Scholar and Embase. The databases were searched for articles published from 2010 to 2021. In addition, grey literature was searched through the “New York Academy of medicine grey literature reports”.^1^ Websites of United States Centers for Disease Control and prevention (US CDC), Africa Centres for Disease Control and Prevention (Africa CDC) and the World Health Organization (WHO) were searched for relevant guidelines, tools and frameworks. We also reviewed the references of retrieved studies to identify additional articles. We chose key terms and developed a search strategy based on the National Library of Medicine Subjects Headings (MeSH)”.

The following search strategy was applied in the PubMed database: “[cholera (Title/Abstract)] AND surveillance [Title/Abstract], [cholera (Title/Abstract)] AND integrated disease surveillance [Title/Abstract], [acute watery diarrhoea (Title/Abstract)] AND surveillance [Title/Abstract], [cholera (Title/Abstract)] AND surveillance performance [Title/Abstract], [acute watery diarrhoea (Title/Abstract)] AND surveillance performance[Title/Abstract], [acute watery diarrhoea (Title/Abstract)] AND surveillance evaluation[Title/Abstract], [cholera(Title/Abstract)] AND surveillance evaluation[Title/Abstract]’’. The PubMed search strategy was adopted for other data bases as well. We limited our search to titles and abstracts of articles.

### Study screening process

First, the selected key words were entered into the database search boxes, and the search was limited to abstracts and titles. After going through the abstract relevant articles where then studied critically for better inside knowledge. The review team was made up of the principal investigator (KM), a systematic review expert (NTF) and two research supervisors (GP and LK) The results of the keywords search were reviewed by two members of the review team (KM and NTF). If the study was judged relevant for the review a third reviewer (LK) conducted the final ascertainment. If the study met the inclusion criteria, it was included in the review. If there was any doubt about meeting the inclusion criteria a decision was made based on the consensus of the review team. Articles unrelated to the aim of the study were excluded. The remaining titles were entered into an excel spreadsheet and sorted.

### Data extraction

The finally included papers were evaluated by two members (GP and LK) of the review team using a data abstraction sheet developed by the research team based on existing strategy and framework for cholera control identified in literature (6, 17, 18) as well as the Joint external evaluation (JEE) and the WHO building blocks of a health. This data sheet included the study variables: Governance legislation and policies (Information flow, cross border collaboration and legislation and policies), Core functions (detection, reporting, feedback, assessment, verification, confirmation), supportive functions (data management, analysis interpretation and use, multi-sectoral one health collaboration, digitalization, Linkage to laboratory capacity, resources, workforce, guidelines and standard operating procedures) and system attributes. If these variables were mentioned in the article, they were included in our data abstraction sheet. If not, the review team used a consensus approach to decide whether the data should be included or not.

### Risk-of-bias assessment

We grouped all literature meeting our criterial for inclusion under three categories (frameworks/guidelines, peer reviewed and non-peer reviewed/grey literature) for improved quality evaluation. We appraised all grey literatures using authority, accuracy, coverage, objectivity, date, significance (AACODS) checklist (19). As part of the selection criteria, a “yes” is assigned for studies meeting all criteria for selection, “partially” if the study largely meets the criterion but differs in some important aspect, “no” if the study differs significantly from the inclusion criterion, “unclear” if the literature provides limited information to consider the paper for inclusion and “NA” (not applicable) if its criterion was not relevant. We also, used the Joanna Briggs Institute (JBI) checklist for systematic review (for peer to peer review literature) to assess the quality of studies in the literature (20). The JBI checklist for qualitative research was also used to assess literature that included qualitative and mixed-method studies. These checklists were used to assess the methodological quality of relevant studies and to determine the extent to which a study has addressed the possibility of bias in its design, conduct and analysis. The risk-of-bias assessment was carried out by two reviewers (KM and NT) and discrepancies were resolved by consensus.

### Data analysis data

We consolidated and summarized results in a tabular form to facilitate the visualization of the availability of key elements evaluated: governance policies and legislation, system core functions, system supportive functions and system attributes. A frequency of distributions, expressed as percentage (%), was calculated for each variable. Analysis was stratified by framework used, core and supportive functions and attributes. The strength and weaknesses of each framework was assessed using the consolidated parameters as a benchmark. The review followed the synthesis without meta-analysis (SWiM) guidelines for the synthesis and reporting of findings extracted from included studies.

## Result

**Figure 1:**
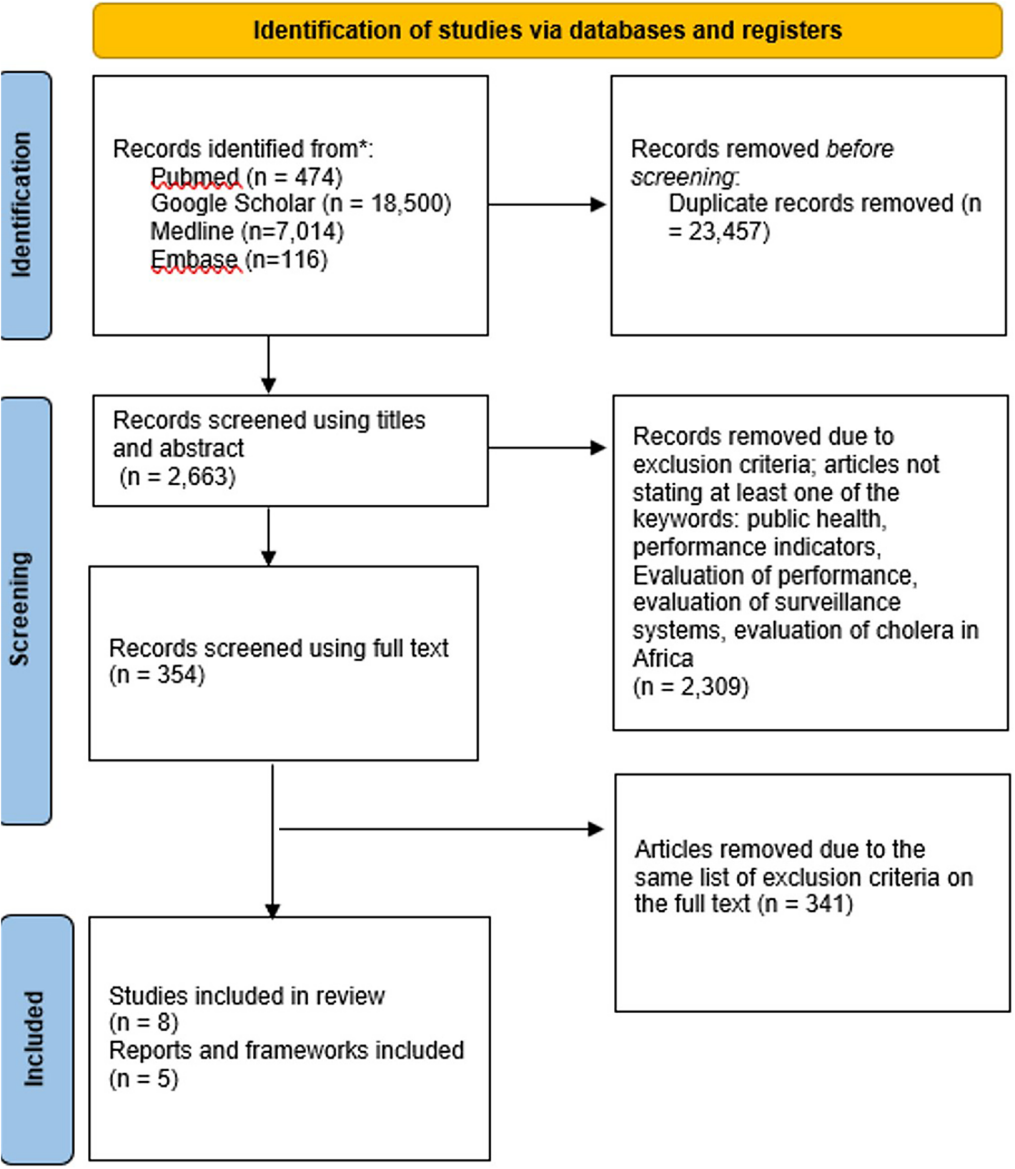
PRISMA flow diagram showing the selection of studies.

Figure 1 shows the PRISMA flow diagram for the selection of studies outlining the literature searches. Of the 13 articles included, five were from grey literature and included frameworks, guidelines, or tools to guide the assessment of surveillance process while eight were research articles on assessment findings. The result of this review highlights the available published tools and also the deployment of some of the tools in conducting assessments of cholera surveillance systems in countries.

### Frameworks, tools and guidelines in reviewed literature

**Figure 2:**
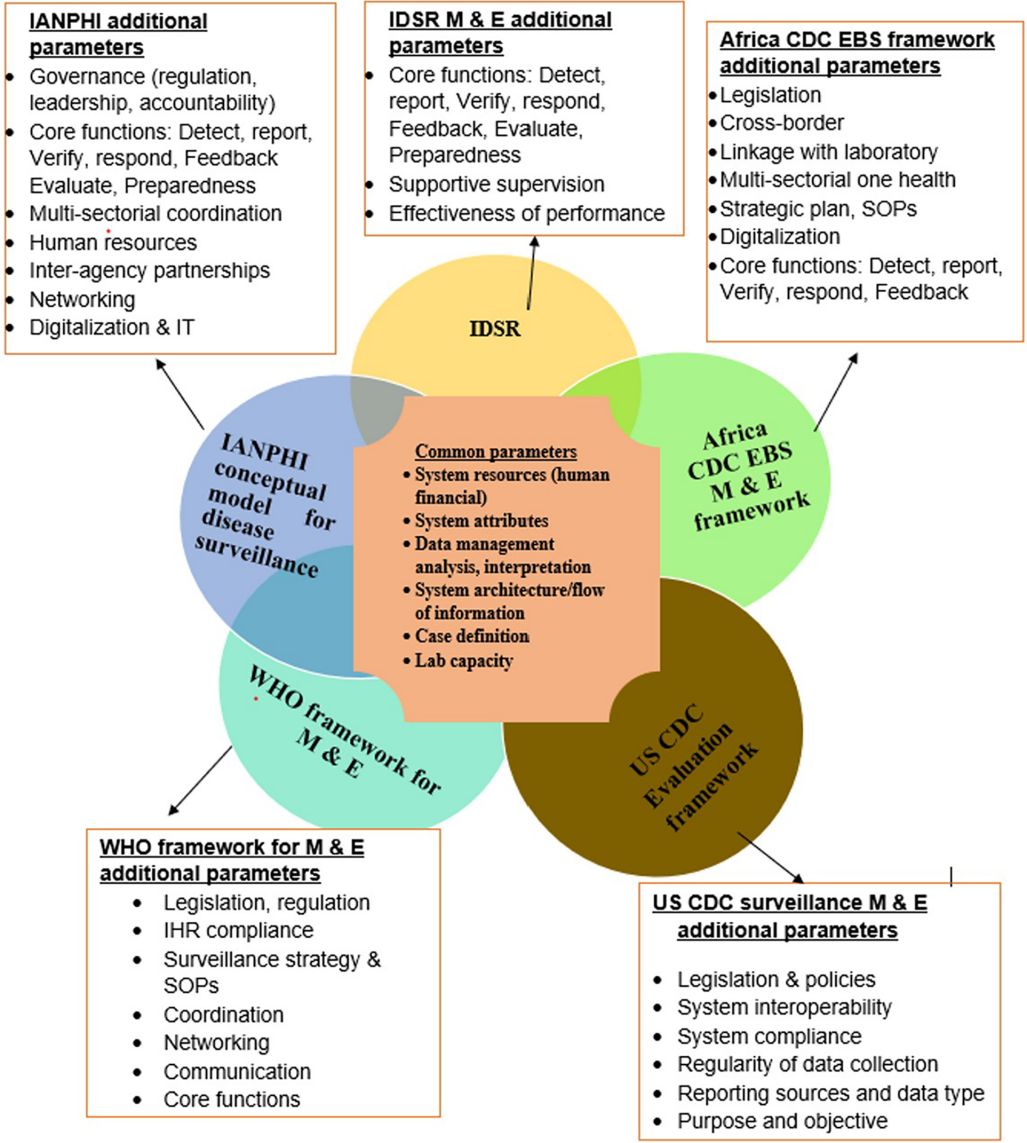
Commonalities and peculiarities of surveillance system assessment framework and tools.

A total of five frameworks/guidelines/tools for communicable disease surveillance evaluation were identified from peer review literature, grey literature, and websites of governmental organizations. These tools include IDSR, Africa CDC EBS framework, the US CDC surveillance monitoring and evaluation framework, the International Association of National Public Health Institutes (IANPHI) integrated disease surveillance report and the WHO framework for monitoring and evaluating surveillance and response systems for communicable diseases. Assessment results show a variation in the strengths and weaknesses across the different frameworks (Table 2). Commonalities and peculiarities of each tool was also assessed to better understand areas of synergy and gaps in the different frameworks (Figure 2).

**Table 2:** Summary of weaknesses and strengths of frameworks/guidelines/tools of surveillance assessements.

Key Assessment constituents	Year of publication	Governance, legislation and policies				Core Functions					Supportive function							Quality and timeliness		
Name of article		Framework used	Legislation and policies	Cross-border collaboration*	Information flow	Case/signal definitions (case detection)	Reporting tools (Case reporting)	Feedback mechanism	Verification and assessment	Lab capacity/confirmation	Data management analysis, interpretation & use	Guidelines and SOPs	Multi sectoral OH coordination	Resources	Workforce (Supervision and training)	Digitalization	Linkage of laboratory systems	System Attributes	Strength	Weaknesses
IANPHI conceptual model	2022	Model	√	**X**	√	√	√	√	√	√	√	√	√	√	√	√	X	√	Considered all core functions of surveillance systems and includes some critical supportive functions such as multi-sectoral coordination, partnership, digitalization and information technology.	Did not consider cross-border collaboration as a parameter to be evaluated. Even though laboratory network was mentioned, it was not clear if linkage of surveillance to laboratory systems was considered
IDSR M and E	2019	Guideline	X	**X**	√	√	√	√	√	√	√	√	**X**	√	√	X	X	√	The Monitoring and evaluation (M and E) tool includes all the core functions in alignment with the IHR (detection, reporting, analysis and interpretation, prepare, respond, feedback, supportive supervision) and also provides a comprehensive list of all system attributes.	Even though the IDSR strategy highlights all core functions and supportive functions, the M and E tool focuses only on the core functions and attributes. Thus, countries may not be able to evaluate critical supportive capacities like multisectoral coordination, cross border, etc
Africa CDC framework and score card	2022	Framework	√	√	√	√	√	√	√	√	√	√	√	√	√	√	√	√	Includes all core functions and supportive functions in the context of event-based surveillance	Countries may not be able to apply some of the indicators in the context of indicator based-surveillance.
WHO M and E framework		Framework	√	**X**	√	√	√	√	√	√	√	√	X	√	√	X	X	√	Includes all core functions and aligns them with the IHR core capacities. Also provides a clear methodology to countries on how to conduct M and E for communicable diseases	Did not include details on cross-border collaboration as part of areas to be considered in the surveillance legislations and governance structures. Also, digitalization and linkage of surveillance to lab capacity was not included.
US CDC	2003	Guideline	√	**X**	√	√	√	X	X	X	√	X	**X**	√	√	X	X	√	Provides a comprehensive picture of the system attributes to be evaluated. It also provides step-wise process to stakeholders on how to conduct an evaluation of a surveillance system.	Majority of the core and supportive functions were not included and thus evaluations done using this tool may provide limited information to stakeholders on areas such as ability of the system to verify events, availability of guidelines and SOPs, multi-sectoral coordination, digitalization etc

X, absent; √, present.

### Review of country cholera surveillance system assessment

Eight articles focusing on cholera surveillance assessment were reviewed. These articles deployed only three of the five available surveillance assessment tools; the US CDC tool for evaluating surveillance systems, WHO framework for monitoring and evaluating surveillance and response systems for communicable diseases and IDSR tools. The studies included a mix of qualitative, quantitative, and mixed methods studies of cholera or integrated disease surveillance (inclusive of cholera surveillance). Amongst the eight primary studies on cholera surveillance assessment, four articles used the US CDC tool in conducting the evaluation while the other four used the IDSR and one used both the IDSR and the WHO monitoring and evaluation framework. All eight articles reported that the surveillance system was meeting their objectives. The parameters used to review the articles were those focusing on legislation, surveillance core and support functions and surveillance attributes (Table 1). Core functions included by the different tools patterning to preparedness and response capacities were excluded; however, linkage of surveillance systems to investigation and response mechanisms was included in the assessment.

### Legislation, governance and information flow

Legislation and governance are cut crossing, but critical components of a functional health system highlighted by the JEE and also the WHO building blocks of a health system. Three of the eight articles mentioned the existence of national and international policies and regulations on surveillance (10). All three articles used the IDSR guideline and highlighted the IHR as the available international legal instrument for the improvement of early detection, disease prevention and response. The IDSR was also mentioned as the national instrument or framework for disease surveillance and response. Six of the eight articles mentioned the assessment of the flow of information across the different administrative levels however; none of the articles mentioned the establishment of cross border surveillance mechanisms or the sharing of cholera alerts across boarders especially in countries where cases were reported in regions or districts bordering other countries.

### Surveillance core functions

All eight articles (100%) mentioned that they evaluated system attributes and some core functions such as detection (availability of case definition for cholera at different levels) and capacity of the system to analyze, manage, interpret and use data. However, other core function such as verification and confirmation (laboratory), reporting and feedback mechanism and tools were not assessed in two of the studies (13, 14). Both studies employed the US CDC surveillance assessment tool. The indicator-based surveillance was found to be dominant over the event-based surveillance. All studies assessed the availability of case definition (to guide detection at health facilities), while the availability of a pre-defined signal list for detecting clusters of diarrheal cases through the event-based surveillance (EBS) approach was not mentioned in all the articles. None of the studies mentioned the evaluation of the use of EBS to monitor food borne diseases (which is one of the core capacities within the JEE). Only a few articles mentioned the use of informal and informal sources in detecting and reporting of suspected cholera cases (11).

### Supportive functions

All the reviewed articles mentioned that assessment of data management, analyses, interpretation and use, however, 50% of the articles did not mention the assessment of the digitalization of the surveillance processes at all levels. All four articles which deployed the IDSR framework assessed the digitalization of surveillance processes at data capturing points and also the availability of digital hardware and solutions to facilitate the process.

All articles safe one, indicated the assessment of resources including financial and human resources (13). In addition, 50% of the articles did not mention the assessment of standard operating procedures at the community, health facilities, sub-national and national levels. Furthermore, none of the articles mentioned the assessment of a functional coordination mechanism for cholera surveillance using a multi-sectoral one-health approach, for cholera response, however, a few articles mentioned the existence of a coordination mechanism and a proposed recommendation for the one health approach to be considered in subsequent outbreak response (10, 15). The linkage of laboratory systems with disease surveillance at the health facility and communities were not assessed in any of the articles, however laboratory capacity to collect and analyze samples was assessed in six of the eight studies.

### System quality and timeliness (surveillance attributes)

The key surveillance attributes used in the different articles include completeness, timeliness, simplicity, acceptability, representativeness, flexibility, usefulness, positive predictive value, sensitivity, data quality, stability and accuracy (Table 3). Of the eight country evaluations reviewed, two didn't document the assessment of the system attributes, however one of the studies assessed the understanding of health care workers on the IDSR indicators which revealed that their knowledge of core indicators was limited to just “completeness” and “timeliness”. Both studies that did not assess the surveillance attributes mentioned using the IDSR tool for assessment.

**Table 3:** Surveillance system attributes assessed in reviewed studies.

Name of article	Assessment objective	Completeness	Timeliness	Simplicity	Acceptability	Representativeness	Flexibility	Usefulness	Positive predictive value	Sensitivity	Data quality	Stability	Accuracy
Cholera public health surveillance in the Republic of Cameroon-opportunities and Challenges. Moise Chi Ngwa et al. (8)	An in-depth description and assessment of the structure, core and support functions, and attributes of the current cholera surveillance system in Cameroon. It also discussed its strengths and challenges with hope that lessons learned could improve the system in Cameroon and in other countries in Africa implementing the IDSR strategy.	√	√	X	X	X	X	X	X	X	X	**X**	X
Evaluation of cholera surveillance system in Osu Klottey District, Accra, Ghana. Eric Yirenkyi Adjei et al. (9)	To assess whether the surveillance system is effective, and also to assess attributes of the system.	X	X	√	√	√	√	√	√	√	X	X	X
A rapid assessment of the implementation of integrated disease surveillance and response system in Northeast Nigeria. Ibrahim et al. (10)	To determine the status of implementation of IDSR in Adamawa, Borno and Yobe states in North-East Nigeria. The assessment also seeks to collect best practices and identify areas for improvement to strengthen the IDSR implementation in the states.	X	**X**	X	X	X	X	X	X	X	X	**X**	X
Evaluation of Cholera and Other Diarrheal Disease Surveillance System, Niger State, Nigeria-2012 Bashorun et al. (11)	To determine how the cholera and other diarrheal disease surveillance system in Niger state is meeting its surveillance objectives, to evaluate its performance and attributes and to describe its operation to make recommendations for improvement.	X	√	√	√	√	√	X	√	√	√	√	X
Evaluation of the integrated disease surveillance and response system for infectious diseases control in northern Ghana. Adokiya et al. (12)	To evaluate the Integrated Disease Surveillance and Response (IDSR) system in northern Ghana.	X	**X**	X	X	X	X	X	X	X	X	**X**	X
Evaluation and use of surveillance system data toward the identification of high-risk areas for potential cholera vaccination: a case study from Niger. Guerra et al. (13)	To provide the results of an evaluation of the surveillance system in the region of Maradi, Niger and an analysis of its data towards identifying high-risk areas that may be considered for potential cholera vaccination.	√	X	X	X	√	X	X	√	√	√	X	√
Assessment of the cholera surveillance system in the Ledzokuku-Krowor municipality in the greater Accra region of Ghana. Achempem, (14)	To assess the cholera surveillance system of the Ledzokuku-Krowor municipality of Ghana, a highly cholera endemic area to determine whether the system is performing effectively.	X	X	X	√	√	√	√	X	X	√	X	X
Evaluation of integrated disease surveillance and response (IDSR) core and support functions after the revitalisation of IDSR in Uganda from 2012 to 2016. Masiira et al. (15)	To assess IDSR core and support functions after implementation of the revitalised IDSR programme.	√	√	X	X	X	X	X	X	X	X	X	X

X, absent; √, present.

## Discussion

Several studies have been conducted on the evaluation of cholera surveillance systems in Africa. However there exist a wide variation in the methodology and tools used in the assessment of these systems. The most important findings from this review include: (a) There is limited information on the availability of specific tools for the assessment of the implementation of cholera surveillance systems; however, several frameworks are available in existing literature to guide the evaluation of systems for communicable diseases and other acute public health threats. (b) The available assessment frameworks vary widely in the recommended parameters for the assessment of surveillance systems. (c) Indicators such as multi-sectoral one health coordination mechanisms; cross border coordination and surveillance systems were not included in any of the cholera surveillance assessments. Even though the surveillance attributes were considered as critical parameter in the assessment of surveillance systems, there were a number of variations in the attributes included even among studies with the same objective (11). Similar studies have demonstrated this variation in the methodology of evaluating surveillance data quality by Member States in the European union which led the European Centers for Disease Control and prevention to develop a standardized framework to guide evaluation of surveillance systems in the region (21).

### Multi-sectoral one health coordination

This parameter was included in the Africa CDC event-based surveillance Monitoring and Evaluation framework and the IANPHI conceptual framework as a critical component for assessment. However, the IDSR indicator list and WHO M & E framework mentioned public health emergency management committees and coordination respectively as indicators but did not include the multi-sectoral one health aspect as an assessment criterion. This variation further trickles down to the country assessments such as that which was conducted in Uganda (15). This assessment revealed that a public health emergency management committee was in place in Uganda, however it was unclear if the multi-sectoral one-health approach was considered in the composition of such a committee. Given that the risk factors of cholera (like poor access to water hygiene and sanitation, overcrowded settings etc) lies outside the health sector, there remains a dire need for the establishment of such multi-sectoral one health coordination mechanism for cholera surveillance and response (22).

### Cross boarder coordination and information sharing

The establishment of cross-border mechanisms of information exchange and coordinated response in border zones was also not considered across the different country assessments conducted. Cholera cases were reported in border regions in Cameroon, Nigeria and Uganda, however there was not any evaluation of information sharing across the borders or cross border coordination surveillance and response mechanisms (23). There has been a growing concern on the increase in cross-border cholera spread in sub-Saharan Africa with a critical recommendation of reviewing guidelines, SOPs and frameworks to integrate the cross-border component to improve information sharing, coordinated surveillance and response especially where there is a risk of cross border spread (22). The Africa CDC EBS and IDSR guidelines highlights cross border coordination and information as a key consideration to consider in controlling acute risk with potential of spread, however, only the Africa CDC EBS scorecard clearly spells out cross-border coordination and information sharing as an indicator to be evaluated during the assessment of the implementation of surveillance system.

### Linkage of surveillance to laboratory systems and networks

This is one of the parameters that even though was very critical, yet was not considered by any of the evaluations. Suspected cholera cases that are detected through the health facilities and communities may require laboratory confirmation to confirm an outbreak in an area. Ensuring synergy and interaction between the public health laboratory systems and disease surveillance systems is critical to improve early detection of cases and control interventions. While the electronic IDSR (e-IDSR) has been widely implemented by several countries, there remain the need to link this electronic platform to other laboratory information management systems for real time information exchange. This would facilitate timely dissemination of information of any emerging health threat for prompt action by public health officials. In addition, a network of laboratory could be established to report to a central data base accessible to public health officials and surveillance officers to support real time data analysis of trends and burden of diseases. Such information could further guide prepared and response efforts like targeted vaccination campaigns and other disease control initiatives.

### Surveillance attributes

One of the parameters also considered by all the frameworks and country system assessments are the surveillance system attributes. While it is important to note that surveillance system attributes were assessed by 75% (*n* = 6) of the assessments conducted in countries, there were still variations in the specific attributes listed by each framework and assessments conducted (Table 3). For the two studies aimed at assessing the IDSR core functions, only the completeness and timeliness were considered in these assessments. Previous studies on the evaluation of surveillance systems have pointed out this gap demonstrating the need for strengthening data collection processes on the continent by harmonizing data collection parameters (24).

## Conclusion

This review highlighted the needs to develop a comprehensive approach for the evaluation of surveillance systems, leveraging existing tools while also incorporating guidance on the assessment of individual parameters. Several organizations have developed evaluation approaches, targeting only partial aspects of the surveillance systems characteristic; and most of the available approaches provide general recommendations for evaluations. This would ensure standardization across countries and would support stakeholders and donors and partners in prioritization and resource allocation. Parameters like cross border, one health multisectoral coordination, availability of digital tools, standard operating procedures and guidelines should be emphasized as critical in the assessment of surveillance systems. In addition, a spectrum of critical attributes should be composed to guide countries in assessing surveillance system performance.

## Recommendations

The following recommendations may be incorporated in the development of a comprehensive surveillance assessment tool:

• Stakeholders should consider assessing the existence of legislations that foster the operationalization of disease surveillance and the exchange of critical information using a one health multisectoral approach. The availability of a clear reporting architecture across the different sectors at different administrative levels could be one of the pieces of evidence to look out for during an assessment.
• Accountability and sustainability mechanisms are some of the capacity gaps to be considered during system assessment. This includes government investment in public health systems including surveillance as well as monitoring and evaluation of short- and long-term targets for health.
• There has been evidence of cross border spread of cholera (23) on the continent emphasizing the need to establish systems to prevent spill overs. Cross boarder coordination mechanisms are critical in controlling cross border spread and improving response efforts for cholera. It is important to understand if legislations, tools and platforms exist to facilitate such initiatives including information exchange across countries.
• It is also important for stakeholders to define a set of critical attributes to be considered across board during surveillance system evaluations. This would support the standardization for such evaluation across countries and outcome could further guide stakeholders to understand gaps and where to tailor resources.
• The need to establish standard operating procedures, plans and processes for surveillance and laboratory systems is pivotal for the harmonization of such procedures within and across countries. This would further strengthen standardization in capacity assessment and resource allocation initiatives in Africa.
• Previous studies (25) have revealed that digitalization improves timeliness in reporting. It would be important for stakeholders to consider including digitalization as a critical parameter when evaluating surveillance systems. This should follow a one-health approach as it is important to also understand how the different sectors are able to share information using interoperable digital solutions.

## Strengths and limitations

The main search was restricted to articles in English. Despite this limitation, databases such as PubMed, Medline and Embase were searched. Grey literature and the website of organizations were also searched, increasing the likelihood of identifying documents that were not published on the conventional databases. A second limitation is the absence of research protocols (data collection tools, interview guides etc.) for each of the articles included in this study. Despite this limitation, the different capacity areas mentioned in the articles were matched with the indicators in the guideline deployed for better analysis. Importantly, this review is one of the few to assess frameworks for cholera surveillance in Africa and the findings will play a critical role in enhancing cholera surveillance in the continent.

## Data Availability

The original contributions presented in the study are included in the article/Supplementary Material, further inquiries can be directed to the corresponding author.

## Footnotes

^1^That which is produced on all levels of government, academics, business and industry in print and electronic formats, but which is not controlled by commercial publishers”.
